# Mental health antecedents in first episode of psychosis: impact on prognosis across transitional age

**DOI:** 10.1007/s00787-025-02880-w

**Published:** 2025-10-27

**Authors:** Lorenzo Pelizza, Fabio Catalano, Emanuela Leuci, Emanuela Quattrone, Derna Palmisano, Simona Pupo, Giuseppina Paulillo, Clara Pellegrini, Pietro Pellegrini, Marco Menchetti

**Affiliations:** 1 Department of Biomedical and Neuromotor Sciences, Alma Mater Studiorum – Università di Bologna, Bologna (BO), Italy; 2 Department of Mental Health and Pathological Addiction, Azienda USL di Parma, Parma (PR), Italy; 3 Pain Therapy Service, Department of Medicine and Surgery, Azienda Ospedaliero-Universitaria di Parma, Parma (PR), Italy; 4 Istituto di Psichiatria “Paolo Ottonello” - via Pepoli, Bologna (BO), 5 - 40126 Italy

**Keywords:** First episode psychosis, Antecedents, Early intervention in psychosis, Outcome, Help-seeking behavior

## Abstract

The beneficial effects of reducing the duration of untreated psychosis on longitudinal outcomes has led to implement early intervention programs during prodromal phase, especially for young people. However, little is known about psychiatric antecedents in people experiencing First Episode Psychosis (FEP). This study aimed (1) to calculate the proportion of FEP participants with previous contact with Child/Adolescent or/and Adult Mental Healthcare Services (CAMHS/AMHS) recruited within a specialized “Early Intervention in Psychosis” service, and (2) to compare sociodemographic, clinical, and treatment parameters between FEP patients with and without psychiatric antecedents across a 2-year follow-up period. At baseline, all participants (aged 12–35 years) completed the Health of the Nation Outcome Scale (HoNOS). A mixed-design ANOVA and a Kaplan-Meier survival analysis were used. The prevalence of antecedents in our FEP population was 48%. 15% had previous contact with CAMHS and only 21% of them experienced care continuity transitioning to AMHS. The most common past diagnoses in the FEP/CAMHS subgroup were conduct disorder (43.4%) and learning disorder (26.3%). Differently, the FEP/AMHS subgroup more frequently had personality disorder (50.8%) and anxious-depressive disorder (35.9%). FEP/CAMHS individuals had higher baseline HoNOS “Psychiatric symptoms” factor score and received higher total number of family psychoeducation sessions than the other subgroups. Our results suggest the importance of enhancing strategies for a better transition for adolescents. Indeed, this population appears to be at risk for higher psychiatric symptoms detected with HoNOS when developing psychosis.

## Introduction

The presence of prodromal phase is extremely common in the development of psychosis and worthy of clinical interest [1]. Cognitive disturbances, social isolation, daily functioning impairment, anxiety, and depression are frequently reported before a First Episode Psychosis (FEP) [2]. In this respect, a recent meta-analysis on this topic reported that, prevalence rate of prodromal states in young people at psychosis onset was 78.3% [3]. In particular, most of people experiencing prodrome (approximately 80%) started seeking help before the age of eighteen (mean age at first specialist contact for mental health problems = 12 years) and were more likely to have previous diagnosis of neurodevelopmental disorders, such as conduct disorder, attention deficit hyperactivity disorder (ADHD), and intellectual disability [4]. Moreover, patients with early onset psychosis (i.e., age < 18 years) showed longer duration of both untreated illness and psychosis. In this regard, a recent meta-analysis also showed that attenuated psychotic symptoms tend to last for more than 1 year before presentation at specialized services for Clinical High Risk of Psychosis (CHR-P) [5]. Therefore, enhancing early help-seeking is important for early intervention in psychosis, especially for decreasing the “Duration of Untreated Psychosis” (DUP) [6].

Another clinically relevant point to consider is that individuals without full-blown psychotic symptoms at the initial assessment may be subsequently diagnosed with non-affective or affective psychosis during a 4-year follow-up period [7]. Indeed, some people could require more time to feel sufficiently secure to disclose their symptomatology or define these experiences as abnormal. On the other hand, clinicians do not seem to timely identify the development of psychosis when treating other disorders [8].

Early intervention programs for psychosis in child/adolescent and adult mental healthcare services have proven to be feasible, clinically relevant, and recommended, specifically in the age group with a high risk of falling through the child/adult service gap [6]. In this respect, the results of a recent study confirmed that 46.8% of individuals dropped out of care as they approached child and adolescent mental health services (CAMHS) transition, and commonly experiencing discontinuity of care during this critical period [9]. Moreover, the risk of service disengagement increases with shorter contact time in CAMHS, is greater in individuals without pharmacological treatment, and decreases in patients with psychosis, bipolar disorder, eating disorders, and neurodevelopmental disorders [9, 10]. For young people, it was also reported that the risk of drop-out decrease with higher scores on the Health of the Nation Outcome Scale (HoNOS) and more severe psychopathology, making the transition to adult mental health services (AMHS) more likely as well as staying in CAMHS than having care discontinuation [11, 12].

However, little is known about people experiencing FEP who have had psychiatric antecedents (especially a prior contact with CAMHS), as well as about a potential protective effect for care continuity through CAMHS/AMHS transition. In this respect, some studies showed interesting clinical and functioning differences between adolescent and adult-onset psychosis. Indeed, compared to adolescents, FEP adults were significantly associated with better functioning, poorer medication adherence, more psychiatric hospitalization, earlier remission of positive symptoms, and better functional recovery [13–15]. Therefore, the *aims* of the current investigation were: (1) to calculate prevalence rates of young FEP people treated within an EIP program previously had been help-seeking in CAMHS or AMHS at the prodromal stage, and (2) to longitudinally compare sociodemographic, clinical, and treatment response parameters between FEP patients with and without psychiatric antecedents across a 2-year follow-up study. Additional aim was also to quantify the care continuity from first mental healthcare service access for psychiatric antecedent to a specialized “Early Intervention in Psychosis” (EIP) program.

## Methods

### Setting and participants

All participants consecutively enrolled within the “Parma Early Psychosis” (Pr-EP) protocol between January 2013 and December 2022 were included in this study. This protocol is a specialized EIP program implemented across adolescent and adult mental healthcare services in the Parma Department of Mental Health (Northern Italy) [16].

*Inclusion criteria* were: (a) specialist help-seeking request, (b) enrollment in the Pr-EP program, (c) age 12–35 years, (d) presence of FEP within one of the following DSM-5 diagnoses: schizophrenia, bipolar disorder or major depressive disorder with psychotic features, delusional disorder, brief psychotic disorder, schizophreniform disorder, and psychotic disorder Not Otherwise Specified (NOS) [17], and (e) a “Duration of Untreated Psychosis” (DUP) of < 2 years. The DUP was defined as the time interval (in months) between the onset of overt psychotic features and the first antipsychotic prescription [18]. This DUP length was selected in accordance to the time limit more commonly used to provide effective interventions within the EIP paradigm [19].

*Exclusion criteria* were: (a) history of previous psychotic episodes (i.e., outside the current illness episode), (b) known intellectual disability (IQ < 70), and (c) neurological or other medical disease with psychiatric symptoms. Past exposure to antipsychotic medication (i.e., at any dosage and in previous illness episode before the Pr-EP enrollment) was considered as a “functional equivalent” of past psychotic episode [20]. Indeed, the original EIP paradigm psychometrically defined the “psychosis threshold” as essentially that at which antipsychotic medication would probably be started in the common clinical practice [21].

All participants (including minors and their parents) provided their written informed consent for the study participation. The research received local ethical approvals (AVEN Ethics Committee protocol n. 559/2020/OSS*/AUSLPR) and adhered to the 1964 Declaration of Helsinki (and its later amendments).

### Assessment

The presence of FEP at entry was detected using the psychometric criteria of the “Comprehensive Assessment of At-Risk Mental States” (CAARMS), approved Italian version [22]. Moreover, the baseline DSM-5 diagnosis was formulated by a minimum of two trained PARMS team members using the Structured Clinical Interview for DSM-5 mental disorders (SCID-5) [23].

The clinical and functioning outcome *assessment* included the Global Assessment of Functioning (GAF) scale^13^, and the Health of the Nation Outcome Scale (HoNOS) [24].

The *GAF* is a widely used scale for the assessment of daily functioning in individuals with severe mental illness, including young people at CHR-P [25].

The *HoNOS* was specifically developed to assess social and clinical outcomes in people with severe mental illness, including early psychosis [26]. As proposed by Wing and co-workers [27], we considered four main outcome domains: psychiatric symptoms, impairment, social problems, and behavioral problems.

Trained PARMS team members conducted both at baseline and every 12 months the clinical and functioning outcome assessment during the 2-year follow-up period. A sociodemographic/clinical chart was also completed at presentation, capturing a broad range of parameters including gender, age at entry, years of education, employment and migrant status, past specialist contact and previous psychiatric antecedents, current substance abuse, DUP, hospitalization, suicide attempt, pharmacological therapy, and specialized psychosocial intervention.

### Procedures

After CAARMS and SCID-5 interviews, FEP participants were divided into FEP + or FEP- subgroups depending on having or not a past specialist contact for mental problems. Information on psychiatric antecedents was collected directly by patients and/or their family members, and/or obtained from medical records. The FEP + group was also dichotomized into FEP/CA and FEP/A subsamples depending if patients have had previous contact with CAMHS or AMHS.

All participants were tested for clinical outcomes with HoNOS and GAF scale both at baseline and along the 2-year follow-up period. Moreover, 2-year incidence rates of service disengagement, new hospitalization, and new suicide attempt were also calculated. As for specialized treatment, the Pr-EP program provided a 2-year comprehensive package including a psychopharmacological therapy and a multi-component psychosocial intervention (combining individual psychotherapy based on cognitive-behavioral approach, psychoeducational sessions for family members, and a recovery-oriented case management) in accordance with the current EIP guidelines [28, 29]. Specifically. low-dose atypical AP medication was used as first-line treatment [30, 31]. Selective serotonin re-uptake inhibitor and benzodiazepine could be prescribed in case of mood changes, anxiety, or insomnia [32]. Individual psychotherapy was developed on cognitive-behavioral modules for psychotic disorders [33]. Family intervention was based on cognitive-behavioral model for early psychosis [34]. Finally, each participant/family had a dedicated case manager to provide an early recovery-oriented rehabilitation and to coordinate all interventions planned [35, 36].

### Statistical analysis

Collected data were analyzed using the Statistical Package for Social Science (SPSS) 15.0 for Windows [37]. All tests were two-tailed with a significance level set at 0.05. There were no missing data.

In between-group comparisons, the Chi-squared (Χ^2^) test for categorical variables and the Kruskal-Wallis test (with the Mann-Whitney test as post-hoc procedure) for continuous measures were used. All p values were corrected for multiple comparisons. As for outcome parameters that had as endpoint the time when a specific event occurs, we performed Kaplan-Meier survival analyses to consider the different duration of follow-ups and participants who dropped out from the study protocol. Finally, a mixed-design ANOVA analysis was performed to evaluate the temporal stability of HoNOS and GAF scores within and between the three FEP subgroups across the 2-year follow-up period.

## Results

**Fig. 1:**
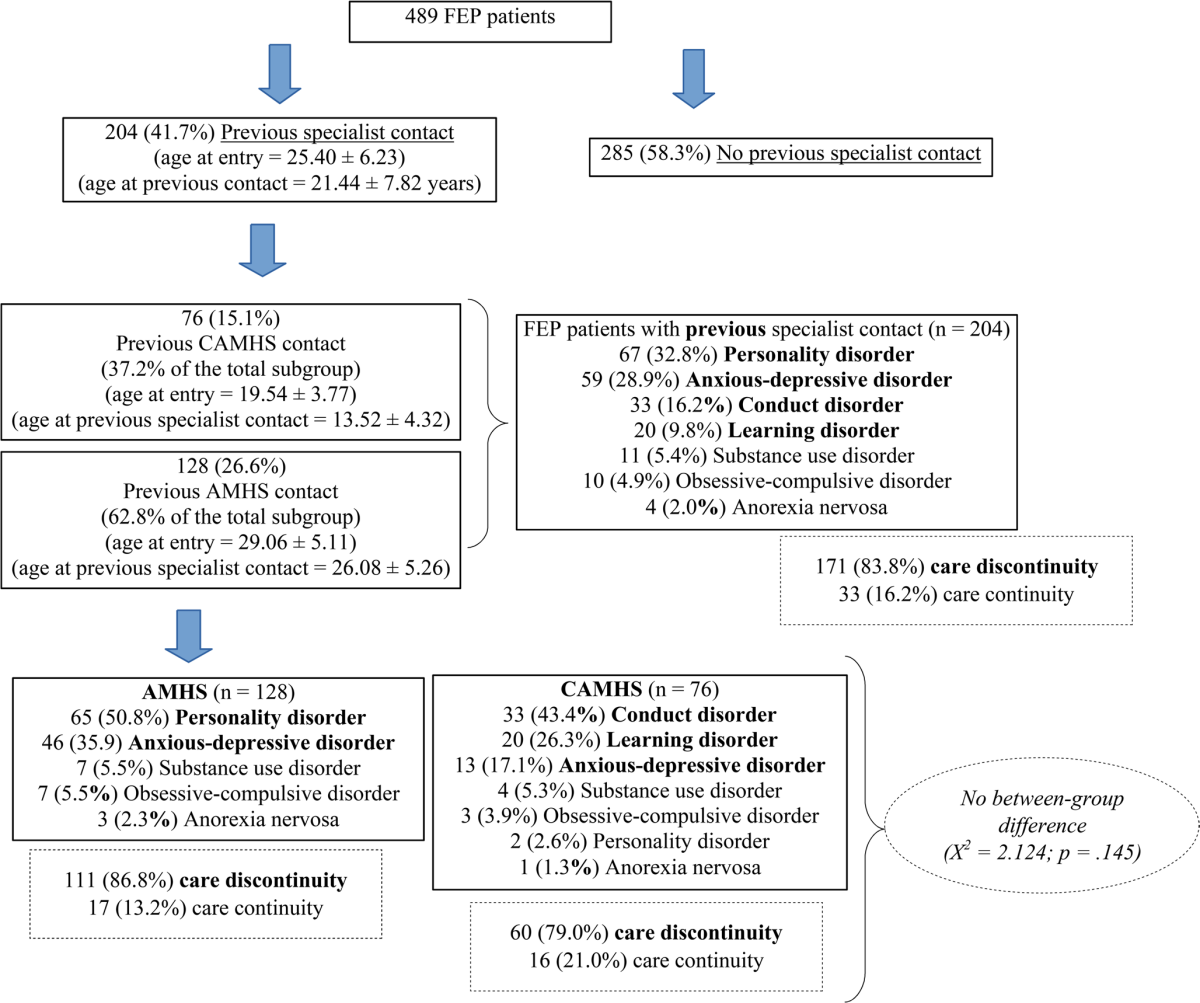
Prevalence rate of FEP patients with and without previous specialist contact (*n* = 489). Note. FEP = First Episode Psychosis, CAMHS = Child and Adolescent Mental Health Services, AMHS = Adult Mental Health Services; X2 = Chi-square test value; p = statistical significance

A total of 489 FEP patients were enrolled in this investigation. Of them, 204 (41.7%) had a previous specialist contact, while 285 were grouped in the FEP- subsample. Specifically, 76 (15.1% of the FEP total population) had past contact with CAMHS (FEP/CA subgroup) and 128 (26.6%) with AMHS (FEP/A subgroup) (Fig. 1).

For FEP/CA participants, age at entry was 19.54 ± 3.77 years and age at previous contact was 13.52 ± 4.32 years (Table 1). For FEP/A individuals, age at presentation was 29.06 ± 5.11 years and age at past specialist contact was 26.08 ± 5.26 years. The main DSM-5 primary diagnoses at the previous contact were: personality disorder (32.8%), anxious-depressive disorder (28.9%), conduct disorder (16.2%), and learning disorder (9.8%). Specifically, the most common past diagnoses in the FEP/CA subgroup were: 43.4% conduct disorder, 26.3% learning disorder, and 17.1% anxious-depressive disorder. Differently, the FEP/A subgroup more frequently had personality disorder (50.8%) and anxious-depressive disorder (35.9%). Only 33 FEP participants (16.2% of the FEP subgroup with previous contact) were directly referred to the Pr-EP program by other mental healthcare services within a clinical pathway of care continuity. In the remaining 171 (83.3%) FEP subjects, their past specialist contact ended with their care retention in mental health services being terminated (i.e., care discontinuity). In particular, 16 FEP/CA individuals (21.0% of the FEP/CA subgroup) experienced care continuity through the transition between CAMHS and AMHS.

**Table 1:** Sociodemographic and clinical comparisons in the three FEP subgroups

Variable	FEP/CA (*n* = 76)	FEP/A (*n* = 128)	FEP- (*n* = 285)	X^2^	*p*	Post hoc
Gender (males) Ethnic group (white Caucasian) Migrant Status Age (at entry) Education (in years) *Civil status* Single Married/partnership *Living status* Alone	42 (55.3%) 66 (86.8%) 8 (10.5%) 19.54 ± 3.77 10.84 ± 1.93 67 (82.2%) 9 (11.8%) 0 (0.0%) 66 (86.8%)	84 (65.6%) 108 (84.4%) 20 (15.6%) 29.06 ± 5.11 11.57 ± 3.11 91 (71.1%) 37 (28.9%) 11 (8.6%) 104 (81.3%)	179 (62.8%) 232 (81.4%) 52 (18.2%) 25.32 ± 5.99 11.62 ± 2.93 182 (63.9%) 102 (35.8%) 22 (7.7%) 223 (78.2%)	2.237 1.483 2.681 119.131 8.010 8.565 7.952 6.619 2.896	0.327 0.476 0.262 **0.001** 0.054 **0.009** **0.015** 0.111 0.235	– – – FEP/CA < FEP-< FEP/A FEP/CA > FEP/A = FEP- FEP/CA < FEP/A = FEP- – – –
Living with partners Living with parents *Occupation* NEET Student *Source of referral* Primary care Family members Self-referral	10 (13.2%) 30 (39.5%) 39 (51.3%) 24 (31.6%) 8 (10.5%) 8 (10.5%) 14 (18.4%) 6 (7.9%) 16 (21.1%)	13 (10.2%) 79 (61.7%) 15 (11.7%) 49 (38.3%) 15 (11.7%) 10 (7.8%) 35 (27.3%) 2 (1.6%) 17 (13.3%)	40 (14.0%) 150 (52.6%) 68 (23.9%) 86 (30.2%) 26 (9.1%) 18 (6.3%) 105 (36.8%) 17 (6.0%) 33 (11.6%)	1.190 49.503 40.364 2.682 0.686 1.611 10.820 4.965 4.619	0.552 **0.027** **0.001** 0.262 0.710 0.447 .**012** 0.084 0.099	FEP/A = FEP->FEP/CA FEP/CA > FEP/A = FEP- – – – FEP->FEP/A = FEP/CA – – FEP/A = FEP/CA > FEP-
Emergency room School/Social services Other mental health services DUP (in months) Previous hospitalization Previous mental health compulsory treatment Previous suicide attempts Substance misuse at entry *Treatments*	11.87 ± 12.48 29 (38.2%) 8 (10.5%) 5 (6.6%) 33 (43.4%) 66 (86.8%) 3.08 ± 3.07 9 (11.8%) 6 (7.9%)	12.23 ± 9.91 60 (46.9%) 13 (10.2%) 3 (2.3%) 41 (32.0%) 110 (85.9%) 2.87 ± 2.54 23 (18.0%) 23 (18.0%)	8.28 ± 8.85 124 (43.5%) 29 (10.2%) 18 (6.3%) 114 (40.0%) 241 (84.6%) 2.96 ± 2.57 57 (20.0%) 37 (13.0%)	23.818 1.475 0.009 3.053 3.311 0.309 0.132 2.688 4.300	**0.001** 0.478 0.996 0.217 0.191 0.857 0.936 0.261 0.116	– – – – – – – – –
Baseline AP prescription Equivalent dose of risperidone (mg/day) Baseline AD prescription Baseline MS prescription Baseline BDZ prescription Acceptance of individual psychotherapy Total number of individual psychotherapy sessions Acceptance of family psychoeducation Total number of family psychoeducation sessions	23 (30.3%) 56 (73.7%) 15.38 ± 14.80 53 (69.7%) 9.65 ± 7.67 56 (73.7%) 35.81 ± 35.06 47 (61.8%) 15 (19.7%)	35 (27.3%) 99 (77.3%) 12.83 ± 12.95 76 (59.4%) 5.83 ± 6.03 105 (82.0%) 34.82 ± 27.00 69 (53.9%) 36 (28.1%)	110 (38.6%) 217 (76.1%) 14.37 ± 14.35 165 (57.9%) 7.12 ± 8.05 206 (76.3%) 31.20 ± 29.33 136 (47.7%) 89 (31.2%)	5.628 0.353 1.252 3.550 8.941 4.575 7.039 5.182 3.899	0.060 0.838 0.535 0.169 .**033** 0.102 0.089 0.075 0.142	– – – FEP/CA > FEP-=FEP/A ––––––– –––––––FEP/CA > FEP/A = FEP- – – – – FEP/CA > FEP-
Acceptance of case management Total number of case management *Baseline DSM-5 diagnosis* Schizophrenia Affective psychosis Brief psychotic disorder Psychotic disorder NOS Schizophreniform disorder *Baseline PANSS scores*	9 (11.8%) 5 (6.6%) 0 (0.0%) 18.82 ± 5.78 22.23 ± 8.45 21.73 ± 7.94 16.14 ± 5.64 9.98 ± 4.61 92.61 ± 22.75	16 (12.5%) 5 (3.9%) 2 (1.6%) 17.53 ± 6.23 24.62 ± 8.10 20.99 ± 7.86 15.09 ± 5.40 10.18 ± 4.56 91.47 ± 23.56	44 (15.4%) 11 (3.9%) 5 (1.8%) 17.61 ± 6.20 24.78 ± 9.08 20.72 ± 7.43 16.55 ± 5.44 9.68 ± 4.99 92.58 ± 23.92	1.011 1.143 1.133 1.915 3.482 0.871 3.490 1.341 0.399	0.603 0.565 0.514 0.384 0.175 0.647 0.175 0.511 0.819	
Positive Symptoms Negative Symptoms Disorganization Affect Resistance/Excitement PANSS total score Baseline GAF score *Baseline HoNOS scores* Behavioral problems Impairment Psychiatric symptoms Social problems Total score 2-year service disengagement incidence rate 2-year new hospitalization incidence rate 2-year new attempted suicide incidence rate	42.47 ± 9.90 3.50 ± 2.35 2.95 ± 1.84 11.37 ± 2.90 7.30 ± 3.66 25.12 ± 7.09 19 (25%) 21 (27.6%) 7 (9.2%)	45.81 ± 9.74 3.67 ± 2.59 3.16 ± 2.12 9.85 ± 2.91 7.80 ± 3.77 24.49 ± 8.29 28 (21.9%) 31 (24.2%) 5 (3.9%)	44.69 ± 10.81 3.94 ± 2.44 3.28 ± 2.18 9.98 ± 3.55 7.76 ± 4.01 24.95 ± 9.16 80 (28.1%) 63 (22.1%) 9 (3.2%)	3.442 2.637 0.923 12.339 0.959 0.485 1.807 1.066 5.412	0.179 0.268 0.630 **0.006** 0.619 0.785 0.405 0.587 **0.045**	

Note. FEP = First Episode Psychosis; FEP/CA = FEP patients with previous specialist contact in Child/Adolescent mental health services; FEP/A = FEP patients with previous specialist contact in Adult mental health services; FEP- = FEP patients without previous specialist contact; NEET = Not in Education, Employment, nor Training; DUP = Duration of Untreated Psychosis; AP = Antipsychotic medication; LAI-AP = Long-Acting Injection Antipsychotic medication; AD = Antidepressant medication; MS = Mood Stabilizer; BDZ = Benzodiazepine; DSM-5 = Diagnostic and Statistical Manual of mental disorders − 5th Edition; PANSS = Positive And Negative Syndrome Scale; GAF = Global Assessment of Functioning; HoNOS = Health of the Nation Outcome Scale. Frequencies (and percentages) and mean ± standard deviation are reported. Chi-square (X^2^) test values are reported. Bonferroni’s corrected p values are reported. Statistically significant p values are in bold

Sociodemographic and clinical comparisons among the three FEP subgroups are summarized in the Table 1.

At baseline, FEP patients with previous specialist contact were younger than FEP- participants, with the lowest age at entry found in the FEP/CA subgroup. Moreover, FEP/CA individuals were more likely to be single, and students than the other two subgroups, and were less likely to be NEET (i.e., “Not in Education, Employment, nor Training”). Furthermore, FEP participants with previous specialist contact had longer DUP than FEP- subjects, who differently were more frequently referred to the Pr-EP program by emergency room. Finally, FEP/CA individuals had higher baseline HoNOS “Psychiatric symptoms” factor score and received higher total number of family psychoeducation sessions than the other two FEP subgroups.

**Fig. 2:**
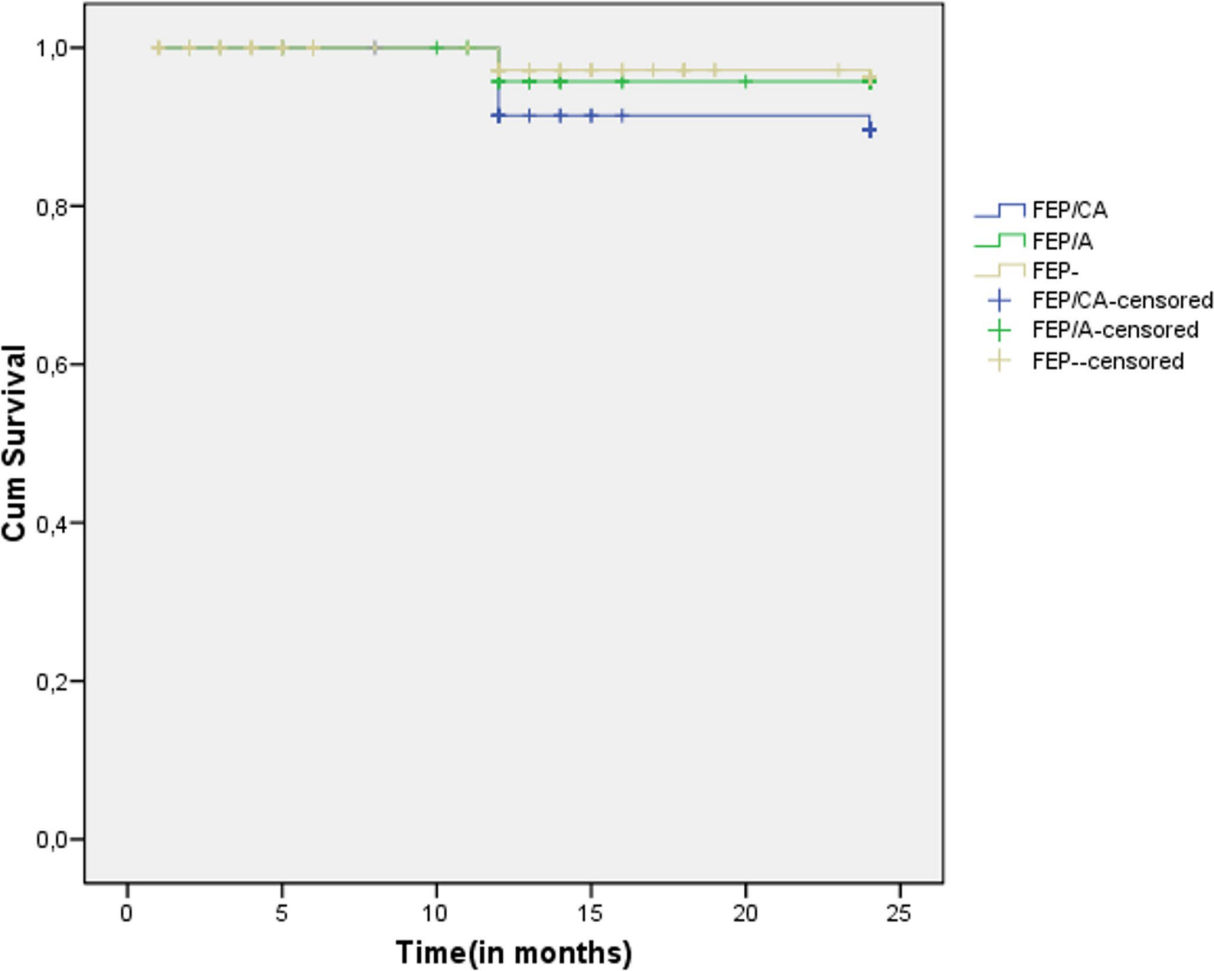
Survival functions: comparison on 2-year new suicide attempt incidence rate among the three FEP subgroups. Note. FEP = First Episode Psychosis; FEP/CA = First Episode Psychosis with previous specialist contact in Child/Adolescent mental health services; FEP/A = First Episode Psychosis with previous specialist contact in Adult mental health services; FEP- = First Episode Psychosis without previous specialist contact

As for clinical outcome, a higher 2-year incidence rate of new attempted suicide across the follow-up was found in the FEP/CA subgroup compared to the other two FEP subsamples. This finding was confirmed also using Kaplan-Meier survival analyses (see Table 2; Fig. 2 for details). No intergroup difference was observed in terms of service disengagement and new hospitalization over time.

**Table 2:** Kaplan-Meier survival analysis results: comparison on 2-year outcome incidence rate among the three FEP subgroups

FEP subgroup	1-cumulative proportion surviving at the time		Mean (in months) for 2-year drop-out rate			
	Estimate	SE	Estimate	SE	95% CI	
					Lower bound	Upper bound
FEP/CA FEP/A FEP- (Overall)	0.250 0.219 0.281 -	0.050 0.037 0.027 –	20.737 21.141 20.007 20.417	0.678 0.510 0.414 0.296	19.407 20.142 19.195 19.837	22.067 22.140 20.819 20.998
Log Rank (Mantel-Cox)			Χ^2^	df	p	
			2.033	2	0.362	
FEP subgroup	1-cumulative proportion surviving at the time		Mean (in months) for 2-year new hospitalization rate			
	Estimate	SE	Estimate	SE	95% CI	
					Lower bound	Upper bound
FEP/CA FEP/A FEP- (Overall)	0.348 0.303 0.294 –	0.061 0.045 0.031 –	23.536 23.806 23.684 23.694	0.270 0.139 0.114 0.086	23.007 23.534 23.461 23.526	24.065 24.077 23.908 23.862
Log Rank (Mantel-Cox)		Χ^2^		df	p	
		0.734		2	0.693	
FEP subgroup	1-cumulative proportion surviving at the time		Mean (in months) for 2-year new suicide attempt rate			
	Estimate	SE	Estimate	SE	95% CI	
					Lower bound	Upper bound
FEP/CA FEP/A FEP- (Overall)	0.123 0.049 0.042 –	0.043 0.021 0.014 –	23.904 23.907 24.000 23.920	0.071 0.103 0.001 0.047	23.764 23.705 24.000 23.828	24.044 24.109 24.000 24.012
Log Rank (Mantel-Cox)			Χ^2^	df	p	
			5.146	2	**0.049**	

Note. *FEP* First Episode Psychosis, *FEP/CA* First Episode Psychosis with previous specialist contact in Child/Adolescent mental health services, *FEP/A* First Episode Psychosis with previous specialist contact in Adult mental health services, *FEP-* First Episode Psychosis without previous specialist contact, *SE* Standard Error, *95% CI* 95% Confidence Intervals, *Log Rank* Logarithm Rank Test, Chi-Square test, *df* degrees of freedom, *p* statistical value. Bonferroni’s corrected p values are reported. Significant statistical p values are in bold

.

**Fig. 3:**
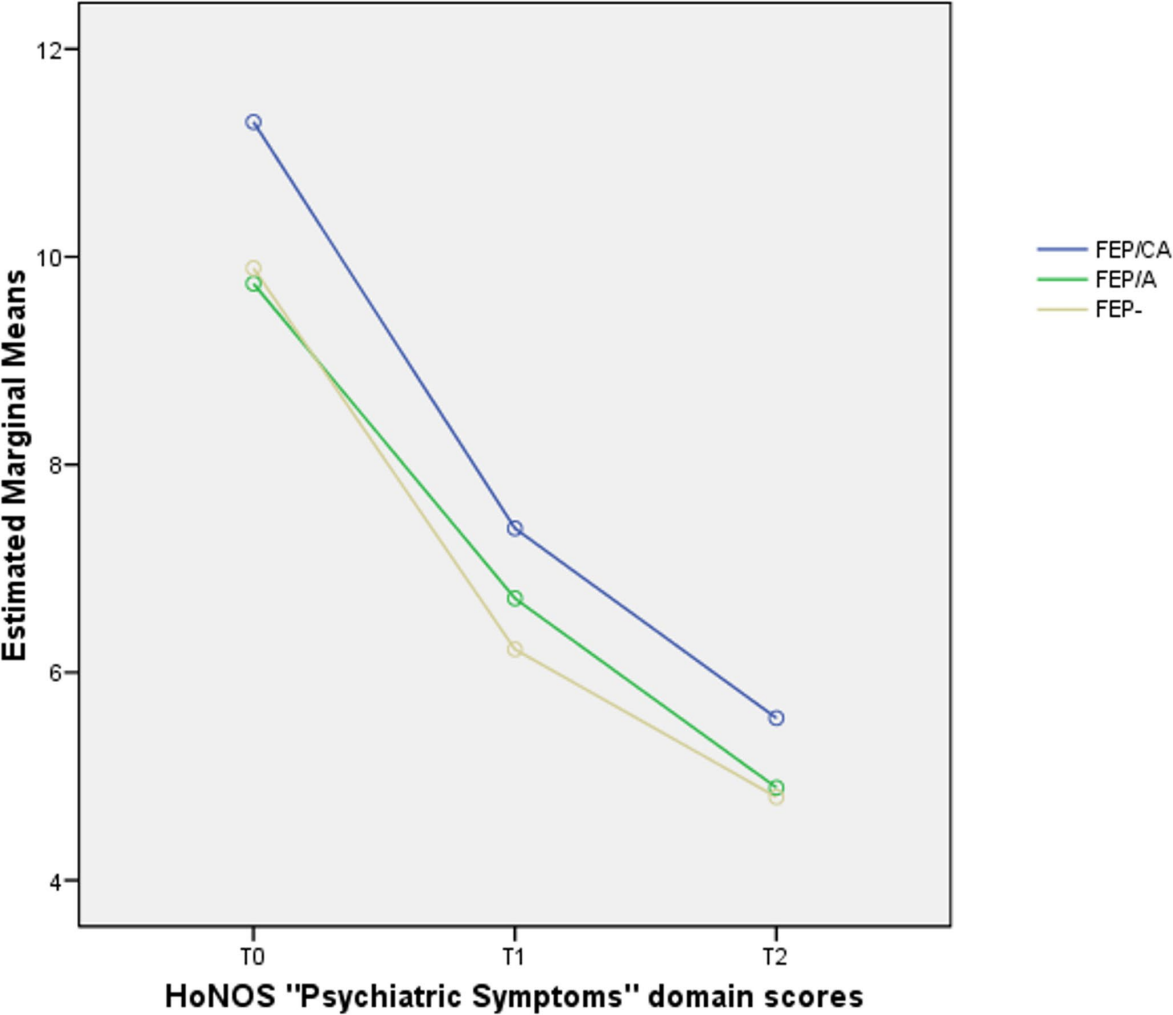
Profile plots: mixed ANOVA results on HoNOS “Psychiatric Symptoms” domain scores across the 2-year follow-up period in the three FEP subgroups. Note - ANOVA = analysis of variance; FEP/CA = First Episode Psychosis with previous specialist contact in Child/Adolescent mental health services; FEP/A = First Episode Psychosis with previous specialist contact in Adult mental health services; FEP- = First Episode Psychosis without previous specialist contact; HoNOS = Health of the Nation Outcome Scale; T0 = baseline assessment; T1 = 1-year assessment time; T2 = 2-year assessment time

Finally, mixed-design ANOVA results showed a statistically significant effect of time on all HoNOS domain scores (Table 3) and a relevant group effect for HoNOS “Psychiatric symptoms” factor subscores in the FEP/CA subgroup (Fig. 3). All partial η² values were higher than 0.140, indicating large effect sizes [38].

**Table 3:** Mixed-design ANOVA results: psychopathological and outcome parameters across the 2-year follow-up period in the three FEP subgroups

Variable	Time effect						Group effect						Interaction effect (time x group)				
	df		F	*p*		η^2^	df		F	*p*		η^2^	df	F		*p*	η^2^
HoNOS Behavioral problems HoNOS Impairment HoNOS Psychiatric symptoms HoNOS Social problems HoNOS Total score GAF PANSS Positive PANSS Negative PANSS Disorganization PANSS Affect PANSS Resistance/Excitement PANSS Total score	1.7 1.5 1.7 1.6 1.6 1.6 1.6 1.7 1.6 1.6 1.4 1.6		198.739 136.926 361.510 170.930 424.159 146.713 210.813 142.971 171.712 171.798 99.505 281.518	**0.0001** **0.0001** **0.0001** **0.0001** **0.0001** **0.0001** **0.0001** **0.0001** **0.0001** **0.0001** **0.0001** **0.0001**		0.352 0.272 0.497 0.318 0.537 0.569 0.479 0.384 0.431 0.429 0.304 0.555	2 2 2 2 2 2 2 2 2 2 2 2		0.204 2.282 3.961 0.854 1.545 0.359 1.397 0.657 0.763 2.283 0.396 0.402	0.815 0.104 **0.020** 0.426 0.215 0.699 0.250 0.519 0.468 0.104 0.673 0.669		0.001 0.012 0.021 0.005 0.008 0.006 0.012 0.006 0.007 0.020 0.003 0.004	3.2 3.0 3.4 3.3 3.2 3.2 3.2 3.4 3.2 3.2 2.9 3.3	1.369 0.728 1.232 1.225 0.528 0.883 0.587 0.923 0.393 1.760 2.140 0.619		0.249 0.536 0.297 0.300 0.673 0.456 0.634 0.438 0.769 0.150 0.098 0.618	0.007 0.004 0.007 0.007 0.003 0.016 0.005 0.008 0.003 0.015 0.018 0.005
Variable		EMM (SE)									Group comparisons				p		
		FEP/CA			FEP/A			FEP-									
T0 HoNOS Psychiatric symptoms T1 HoNOS Psychiatric symptoms T1 HoNOS Psychiatric symptoms		11.298 (0.442) 7.386 (0.454) 5.561 (0.404)			9.743 (0.332) 6.713 (0.341) 4.891 (0.303)			9.891 (0.230) 6.223 (0.236) 4.801 (0.210)			FEP/CA vs. FEP/A FEP/CA vs.FEP- FEP/A vs.FEP-				0.086 **0.016** 0.999		

Note. As all Mauchly’s tests of sphericity are statistically significant (*p* < 0.05), Greenhouse–Geisser corrected degrees of freedom to assess the significance of the corresponding F value are used. Statistically significant p values are in bold. Statistical trends in p value (*p* < 0.01) are underlined. ANOVA = analysis of variance; FEP/CA = First Episode Psychosis with previous specialist contact in Child/Adolescent mental health services; FEP/A = First Episode Psychosis with previous specialist contact in Adult mental health services; FEP- = First Episode Psychosis without previous specialist contact; PANSS = Positive And Negative Syndrome Scale; df = degrees of freedom; F = F statistic value; GAF = Global Assessment of Functioning; HoNOS = Health of the Nation Outcome Scale; p = statistical significance; η^2^ = partial eta squared; EMM = Estimated Marginal Mean; SE = Standard Error; T0 = baseline assessment; T1 = 1-year assessment time; T2 = 2-year assessment time. Bonferroni’s corrected p values are reported. Statistically significant p values are in bold

## Discussion 

Psychiatric antecedents in young FEP population may better inform about prognosis and psychopathological trajectories in young people experiencing psychosis. In this investigation, approximately 42% of FEP participants had a previous contact with mental health services (specifically, 15% of the total sample previously accessed in CAMHS). In contrast with this relevant prevalence of psychiatric antecedents in FEP patients with previous contact in CAHMS or AMHS, care continuity within generalist/first-level mental healthcare centers was very low. These findings are even lower than what was reported in comparable epidemiological studies conducted in different countries (with prevalence rates in retention in care ranging from 36.5% to 58.0%) [39, 40], but similar to another comparable Italian investigation (19%) [41].

Overall, although this investigation mainly focused on FEP patients who had previously sought help, our findings highlight a critical problem for clinical practice: namely, a relevant portion (more than a third) of young FEP individuals seek specialist help for mental distress in a crucial age range, but lack sufficient screening for effective psychosis prevention and a potential identification of an at-risk mental state for psychosis [42]. Among reasons for the complexity behind transition period, there are a significant psychological vulnerability (i.e., higher rates of full-blown mental disorders among late adolescents in comparison with younger teenagers) [43], a lack of appropriate care and ad-hoc services, and a general reluctance of late adolescents to seek help among mental health professionals and services [44]. In this respect, the European “Milestone” survey on the architecture and functioning of CAMHS clearly indicated that the organization of services and the distribution of resources are often not based on young users’ perspectives and needs, as they should be [45]. Such distance from adolescents’ expectations is even more critical as it fails to match the epidemiological burden and the natural pattern of emerging psychosis in young people [46]. Indeed, youths aged 12–25 years have the highest prevalence and incidence of severe mental disease across the lifespan, while also having the worst access to and engagement with psychiatric services compared with all other age groups [47]. Therefore, the division of mental health care along the pediatric/adult model, which was inspired by the traditional organization of somatic medicine, is unfortunate, as it cuts across the age when risk for psychosis peaks, with obvious consequences in terms of service disengagement, under-treatment, discontinuity of care, and unmet needs [48, 49]. In this respect, a recent study on youths’ experiences of moving from CAMHS to AMHS showed that young people are strongly influenced by concurrent life transitions and individual preferences regarding autonomy and independence [50]. Positive factors identified during transition were preparation, flexible transition timing, individualized transition plans, and informational continuity.

For all these reasons, early intervention in psychosis is transitional in nature, bridging the gap between CAMHS and AMHS [51]. In line with McGorry and his team [52], we can no longer postpone a radical review of the structure and resourcing of mental health care for young people in transition from childhood to adulthood. To be sustainable, it is necessary to bridge the serious gap between child-adolescent and adult psychiatry as soon as possible. According to the authors, this might be achievable through a framework shift that incorporates the full continuum of service response within a promotion and prevention framework for youth mental health [53]. Moreover, besides improving the accessibility of youth mental health, also creating “youth-friendly” primary care platforms, another crucial driving principle could be the stepwise gradient of increasing intensity and specificity of treatment, inspired by a developmentally informed clinical staging model [54].

However, our baseline prevalence of past specialist contact is lower than those reported in other comparable epidemiological studies, ranging from 56% to 75% [55, 56]. This difference in prevalence may also be due to differences in the mean age at presentation. Indeed, our FEP population was slightly older compared to samples recruited in previous investigations (25.40 VS. 20.40 years). Overall considered, all these findings confirm how it’s important to pay attention and carefully monitor help-seeking-behavior in young patients typically manifested in their early 20’s, especially in terms of psychosis prevention and intervention [57].

As for primary psychiatric diagnosis at baseline, our findings are substantially in line with what was reported in other studies relatively to past anxious-depressive disorder and past conduct disorder [55, 56], but they differ in terms of higher rates of personality disorder and lower rates of substance use disorder. In this respect, we hypothesized a frequent failure in Italian young FEP participants to honestly admit substance misuse, mainly due to cultural/moral reasons [58]. Moreover, as for previous neuro-developmental disorder having higher prevalence (31.3%) in the study by Ortiz-Orendain and colleagues [55], we observed a 9.8% baseline rate of learning disorder and only 2 FEP participants with past comorbid attention-deficit hyperactivity disorder (ADHD) together with conduct disorder. Examining the main DSM-5 diagnosis in individuals with past contact with CAHMS vs. AHMS, a higher prevalence rate of conduct disorder in the first subgroup was found rather than personality disorder in the second subsample. These finding may be explained by the assumption that personality in adolescence is still not well established and by the spread of conduct disorder through personality disorders [59]. Otherwise, a recent study reported a 5% prevalence rate of people at risk for psychosis who met criteria for DSM-5 conduct disorder and 10% individuals with undetermined conduct symptoms, suggesting that conduct disorder could be a potential differential disease in youth with mental and substance use disorders and psychosis, and that this comorbidity may complicate the outcome of each psychiatric disorder if not well identified and managed [60]. Moreover, it is commonly acknowledged that conduct disorder is the most frequent reason for referral of young children to CAMHS and that it may evolve into antisocial personality disorder in almost half of cases [61]. Otherwise, it was reported that people with schizophrenia who committed a crime and had a previous diagnosis of conduct disorder had more severe positive symptoms and a steadily high-risk assessment score [62], leading to a clinically relevant doubt that their past diagnosis may mean a different suffering for these patients.

As for sociodemographic and clinical characteristics at presentation, FEP/CA participants showed to be younger, more frequently singles and students (as expected), and consequently to be less frequently NEET (despite no intergroup difference in terms of baseline levels of negative symptoms and daily global functioning). Furthermore, FEP patients with previous contact had longer DUP. This appears to be coherent with their low rates of service engagement and care continuity. On the other hand, participants with no previous contact more frequently were referred by emergency room, possibly meaning less familiarity with mental healthcare services and higher fear of stigma [63]. Finally, patients with previous contact with CAMHS had higher number of family psychoeducational sessions, showing a better care engagement of their social support.

As for clinical outcomes, the FEP/CA subgroup notably showed higher levels of HoNOS “Psychiatric symptoms” domain both at baseline and across the 2-year follow-up period, as well as a higher incidence rate of suicide attempt. Overall, these results seem to suggest that FEP/CA participants stably represent the most clinically severe FEP subsample along the follow-up, independently from provided treatments. These findings appear counterintuitive with the common belief that individuals with previous contact with mental health services during childhood or adolescence should have partially contented their psychopathology, their distress and their mental health need through clinical treatment. In this sense, a personal history of previous specialist contact with CAMHS may be considered a clinical index of worsening psychopathology over time (although this relationship has no established causality and other factors may contribute [e.g., higher clinical severity at baseline, greater premorbid functioning decline]). Otherwise, the low rate of care continuity in this subgroup suggests a short-term use of specialist services, potentially aimed at an immediate resolution of symptoms. Overall, one question remains: what are the potential mechanisms underlying the association between prior CAMHS contact and poorer outcomes? Perhaps, ineffective and short-term retention in care during adolescence does not decrease the DUP [64]. Perhaps, a previous non-psychotic diagnosis prevents early detection of FEP [65].

### Limitations

This study had noteworthy limitations. First, information on psychiatric antecedents was collected retrospectively from medical records. This methodology is prone to information bias, as the data in clinical records may not be accurate or complete (especially for diagnosis).

Another limitation concerned that there was no knowledge about potential previous primary care treatments. Indeed, some FEP patients could have received pharmacological treatment for depressive and anxious symptoms from their general practitioner. This may also have resulted in an underestimation of FEP participants with mental health antecedents. Likewise, reliance on past specialist contact information may be subject to recall bias or incomplete records.

Forth, our study was conducted within a specialized “Early Intervention in Psychosis” (EIP) service and this might limit the generalizability of the findings to other settings with different service models or patient populations. Moreover, although our examination compared subgroups, outcome analyses were not specifically matching on potentially confounding variables.

Sixth, FEP patients included in our research were not higher than 35 years. This does not potentially avoid us to consider possible differences in terms of gender. Indeed, it is known that women start with psychosis latter than men (sometimes related with post-partum or motherhood). To better investigate this putative differential factor, future FEP research extending age range beyond 35 years old is therefore needed. Finally, our research was limited to a 2-year follow-up period. Thus, our findings are comparable exclusively with investigations having a longitudinally similar design. Future studies with longer follow-up duration are needed.

### Conclusions

In our study we found out a prevalence of antecedents in a FEP population of 48%. Specifically, 15% of our study group had previous contact with CAMHS but only 21% of them experienced care continuity transitioning to AMHS. This result evidences the importance of enhancing strategies for a better transition for adolescents. Indeed, this population appears to be at risk for higher psychiatric symptoms detected with HoNOS when developing psychosis. Moreover, greater effort should be made to detect psychiatric antecedents of psychosis in both generalist mental healthcare centers and primary care in order to reduce DUP and facilitate service engagement.

## Data Availability

No datasets were generated or analysed during the current study.
